# Vasopressor Dosing Trajectories in Septic Shock According to Initial Antimicrobial Susceptibility and Infection Source Concordance: A Retrospective Observational Study

**DOI:** 10.3390/medsci14030394

**Published:** 2026-07-15

**Authors:** Yurina Yamaya, Tsukasa Kuwana, Kosaku Kinoshita, Ken Takahashi, Hayato Nakabayashi, Toru Imai

**Affiliations:** 1 Division of Emergency and Critical Care Medicine, Department of Acute Medicine, Nihon University School of Medicine, 30-1 Oyaguchi Kami-cho, Itabashi-ku, Tokyo 173-8610, Japan; yamaya.yurina@nihon-u.ac.jp (Y.Y.); kinoshita.kosaku@nihon-u.ac.jp (K.K.); takahashi.ken08@nihon-u.ac.jp (K.T.); nakabayashi.hayato@nihon-u.ac.jp (H.N.); 2 Department of Pharmacy, Nihon University Itabashi Hospital, 30-1 Oyaguchi Kami-cho, Itabashi-ku, Tokyo 173-8610, Japan; imai.toru@nihon-u.ac.jp

**Keywords:** septic shock, norepinephrine equivalent, initial antibiotic therapy, antimicrobial susceptibility, infection source concordance

## Abstract

**Background/Objectives**: Septic shock carries high mortality. Although guidelines recommend early empiric antibiotics, early identification of the infection source and selection of appropriate initial antibiotic therapy remain challenging. This study examined whether vasopressor trajectories, quantified as norepinephrine equivalent (NEE), differed according to initial antimicrobial susceptibility and concordance between presumed and final infection sources. **Methods**: This single-center retrospective observational study included adults with Sepsis-3 septic shock admitted to the intensive care unit between 2017 and 2023. Patients were classified into the appropriate-management group when the initial antibiotic therapy covered the subsequently identified causative pathogen and the initially presumed infection source was concordant with the final infection source; all others were classified into the inappropriate-management group. Primary outcomes were Max NEE duration and the times from Max NEE to 75%, 50%, 25%, and discontinuation of NEE. **Results**: Among 132 patients analyzed, 101 were classified into the appropriate-management group and 31 into the inappropriate-management group. Max NEE duration was shorter in the appropriate-management group (median (IQR), 4 (2–7) h vs. 6 (3–20) h; *p* = 0.0100) and remained shorter after excluding deaths during the Max NEE period (*n* = 119; 6 (2–11) h vs. 11 (5–14) h; *p* = 0.0314). Time to NEE reduction from Max NEE was shorter in the appropriate-management group, with significant differences at 75% (7 (4–13) h vs. 13 (9–23) h; *p* = 0.0066) and 25% (19 (13–29) h vs. 31 (16–46) h; *p* = 0.0247). **Conclusions**: Prolonged Max NEE duration and delayed NEE reduction were associated with inadequate coverage by initial antibiotic therapy or discordant infection source identification in this selected cohort. Vasopressor trajectories may help prompt reassessment of antibiotic therapy and infection source evaluation.

## 1. Introduction

Septic shock is a life-threatening form of circulatory failure with infection-associated organ dysfunction and continues to carry substantial mortality; recent studies have reported a 28-day mortality of 28.9% [1]. Appropriate initial management of septic shock, particularly prompt antibiotic administration, improves survival. Therefore, the Surviving Sepsis Campaign (SSC) guidelines, an international guideline for sepsis management, recommend administering appropriate antibiotics as early as possible, ideally within 1 h [2]. In addition, identification of the infection source and timely source control are essential to improve outcomes. In community-acquired sepsis, source control within 6 h of diagnosis is associated with lower risk-adjusted odds of 90-day mortality [3]. Therefore, rapid initiation of appropriate empiric antibiotics and early identification of the infection source are key components of the initial management of septic shock.

However, in initial clinical practice, empiric antibiotics may be ineffective against the causative bacteria. In addition, the infection source may be misidentified during the initial assessment. In sepsis [4] and bacteremia [5], mortality is higher when initial antibiotic therapy is ineffective because of antimicrobial resistance. Kumar et al. demonstrated that delays in administering effective antibiotics are associated with reduced survival [6]. Furthermore, among patients presenting to the emergency department with infection, an incorrect presumed source of infection at the initial evaluation is associated with increased in-hospital mortality [7]. Although microbiological results, imaging findings, and laboratory markers are essential for reassessing treatment adequacy, they may be unavailable, delayed, or insufficiently specific during the early phase of septic shock management. Biomarker combinations may contribute to sepsis prediction, diagnosis, and estimation of antibiotic treatment duration; however, they currently remain insufficient for determining the appropriateness of initial antibiotic therapy against the causative pathogen [8]. Therefore, readily available bedside information that can complement reassessment of antibiotic therapy and infection source evaluation remains clinically important.

In this context, we focused on vasopressor dosing trajectories in response to septic shock as an observable bedside clinical course that may reflect treatment response. This study therefore evaluated whether the subsequent hemodynamic course differed according to whether the initial antibiotic therapy covered the subsequently identified causative pathogen and whether the initially presumed infection source was concordant with the final infection source. The norepinephrine equivalent (NEE), which enables standardized assessment of different vasopressor doses used for shock management, has recently been updated [9]. Using this NEE, a previous study of patients with septic shock complicated by bacteremia reported that vasopressor doses decreased earlier when the causative bacteria were susceptible to initial empiric antibiotic therapy than when they were resistant [10]. However, bacteremia reportedly accounts for only approximately 28.7% of septic shock cases [11]. Therefore, it remains unclear whether vasopressor dose-reduction trajectories differ according to initial antimicrobial susceptibility in a broader septic shock population, including those without bacteremia. In addition, the relationship between concordance of the presumed and final infection sources and vasopressor dosing trajectories remains unclear.

We hypothesized that vasopressor dosing trajectories would differ according to susceptibility of the causative pathogens to initial antibiotic therapy and concordance between the presumed and final infection sources. The objective of this study was to examine whether vasopressor dosing trajectories differed according to initial antimicrobial susceptibility and infection source concordance in patients with septic shock.

## 2. Materials and Methods

### 2.1. Study Design and Study Population

This single-center retrospective observational study was conducted at Nihon University Itabashi Hospital, a tertiary emergency and critical care center that primarily manages critically ill patients transported directly from the field by emergency medical services, with relatively few interhospital transfers. The intensive care unit (ICU) functions as a mixed medical-surgical unit and does not routinely admit patients after scheduled elective surgery. Adult patients with septic shock who were admitted to the ICU of the Emergency and Critical Care Center between 1 January 2017, and 31 December 2023, were included. Septic shock was defined according to the Sepsis-3 criteria [12]. Patient data were retrospectively extracted from electronic medical records.

Patients were retrospectively categorized into two groups based on a composite assessment of initial antibiotic coverage and infection source concordance. The appropriate group comprised patients whose initial antibiotic therapy covered the subsequently identified causative pathogen and whose presumed source of infection at the initial evaluation matched the final infection source. The inappropriate group comprised patients who met at least one of the following criteria: (1) the causative pathogen was not covered by the initial antibiotic therapy, or (2) the presumed source of infection at the initial evaluation did not match the final infection source. In this study, the terms “appropriate” and “inappropriate” refer only to this retrospective composite classification and do not represent an overall judgment of the quality of initial clinical management. This classification was not intended to evaluate compliance with institutional protocols or the quality of antimicrobial stewardship, but rather to describe whether initial antibiotic coverage and infection source identification were concordant with subsequent microbiological and clinical findings. Susceptibility to initial antibiotic therapy was determined based on antimicrobial susceptibility testing of the causative organism isolated from clinical cultures. The presumed source of infection was defined as the clinically suspected source documented by the treating physician during the initial evaluation. The final infection source was determined at ICU discharge based on the clinical course and the results of microbiological and other diagnostic testing. Susceptibility to initial antibiotic therapy and concordance between the presumed and final infection sources were retrospectively verified by an infectious disease specialist through review of the electronic medical records.

### 2.2. Exclusion Criteria

Patients were excluded if they met any of the following criteria: palliative care only; cardiopulmonary arrest before ICU admission; initiation of antibiotics or vasopressors at another hospital or outside the ICU before ICU admission; death within 24 h after ICU admission; no identified causative pathogen; or an undetermined infection source.

Patients who declined artificial organ support, surgical intervention, or ICU-level treatment were classified as palliative care cases and excluded. Patients who experienced cardiopulmonary arrest before ICU admission were excluded because vasopressor administration after resuscitation may increase substantially independent of septic shock. Patients in whom vasopressor dosing had already been initiated at another hospital or outside the ICU were excluded because the vasopressor dosing trajectory from the time of ICU admission could not be evaluated accurately. Patients who died within 24 h after ICU admission were excluded because the maximum vasopressor dose and the subsequent course of dose reduction could not be assessed reliably. Patients with negative results for all cultures and no identified causative organism, as well as those whose infection source remained undetermined until ICU discharge, were excluded because the two key exposures in this study, susceptibility of the causative pathogen to initial antibiotic therapy and concordance between the presumed and final infection sources, could not be evaluated. Patients with negative blood cultures were not excluded if a causative organism was identified from cultures of other specimens. Consequently, the final cohort consisted of patients in whom both microbiological assessment and infection source classification were available and in whom vasopressor dosing trajectories could be evaluated.

### 2.3. Clinical Protocol

In this ICU, the target blood pressure for patients with septic shock was a mean arterial pressure (MAP) of 65–70 mmHg, in accordance with the SSC guidelines [2]. Within this target range, vasopressor dosing was titrated at the treating physician’s discretion according to the patient’s hemodynamic status. Norepinephrine (NE) was used as the first-line vasopressor. Vasopressin (VA) was added as a second-line agent when an adequate MAP could not be maintained despite an NE dose >0.1 μg/kg/min. Cardiac function was assessed during the initial evaluation, and if depressed cardiac function was suspected, additional vasopressors such as dopamine (DoA) and epinephrine (Epi) were considered. Crystalloid solutions were used for initial resuscitation, and the administered volume was determined in accordance with international guidelines and the patient’s clinical condition. When high-dose vasopressor therapy was required, low-dose corticosteroids (hydrocortisone 200 mg/day) were generally administered [13]. Selection of initial antibiotic therapy and the source control strategy was determined by the critical care physician responsible for the initial management, in accordance with the hospital infection control manual and the suspected infection source, disease severity, patient background, and renal function.

### 2.4. Microbiological Testing

All microbiological cultures and susceptibility testing in this study were performed in the hospital’s in-house microbiology laboratory. During the study period, matrix-assisted laser desorption/ionization time-of-flight mass spectrometry (MALDI-TOF MS) was used for organism identification. Rapid microbiological diagnostic methods, such as multiplex polymerase chain reaction (PCR) assays, as well as rapid susceptibility testing, were not available. In addition, the antimicrobial stewardship team and microbiology laboratory technologists were not available on site 24 h a day.

### 2.5. Sample Collection and Measurements

The following variables were collected from clinical records: age, sex, body mass index (BMI), body weight, ICU survival, ICU length of stay, 28-day survival, Sequential Organ Failure Assessment (SOFA) score, relevant laboratory data, healthcare exposure and risk factors for multidrug-resistant (MDR) organisms including acquisition setting such as community-acquired infection, healthcare-associated infection, or hospital-acquired infection, hospitalization for ≥2 days within the previous 90 days, and prior antibiotic exposure within the previous 90 days, comorbidities, including diabetes mellitus, chronic pulmonary disease, chronic liver disease, chronic kidney disease, chronic heart failure, immunosuppression, and malignancy, and supportive treatments, including mechanical ventilation, renal replacement therapy, low-dose corticosteroids, total fluid volume administered during the first 24 h, extracorporeal membrane oxygenation (ECMO), invasive source control procedures, and details of source control measures including drainage/decompression, surgery/resection/repair, debridement/amputation, and device removal/exchange. Community-acquired, healthcare-associated, and hospital-acquired infections were defined according to standard criteria: hospital-acquired infection was defined as infection diagnosed >48 h after admission and not present at admission; healthcare-associated infection was defined as infection diagnosed ≤48 h after admission when any of the following criteria were met: hospitalization for ≥2 days within the previous 90 days, residence in a nursing home or long-term care facility, maintenance hemodialysis, intravenous chemotherapy, or receipt of home intravenous therapy, wound care, or specialized nursing care within the previous 30 days; otherwise, infections were classified as community-acquired. Prior antibiotic exposure was defined as systemic antibacterial therapy administered within 90 days before the onset of the current septic shock episode. Immunosuppression was defined as systemic corticosteroid use, immunosuppressive therapy, or antineoplastic therapy at the time of septic shock onset. Twenty-eight-day survival was ascertained from in-hospital records; cases without 28-day status reflected transfer to another facility or discharge before day 28, rather than data-collection errors, because post-discharge follow-up was not available. Data were also collected on infection source, including urinary tract infection, pneumonia, intra-abdominal infection, biliary tract infection, skin and soft tissue infection, and other sources; positive culture specimens, Gram-stain-based bacterial classification from blood cultures, including Gram-positive cocci (GPC), Gram-positive rods (GPR), Gram-negative cocci (GNC), and Gram-negative rods (GNR); initial antibiotic therapy; time from septic shock diagnosis to initiation of initial antibiotic therapy; whether antibiotic de-escalation was performed; duration of antibiotic therapy; resistant organism profiles including resistant organism type; and vasopressor dosing. The SOFA score was calculated based on six organ systems: respiratory, coagulation, liver, cardiovascular, central nervous system, and renal, with higher scores (range, 0–24) indicating greater severity and higher risk of ICU mortality [14].

### 2.6. Primary Outcome

The primary outcomes were (1) the duration of the maximum NEE (Max NEE duration), defined as the period during which the highest NEE was maintained, and (2) the time required for NEE to decrease from Max NEE to 75%, 50%, 25%, and 0% (discontinuation). Time zero (0 h) was defined as the time when patients met the Sepsis-3 diagnostic criteria for septic shock [12]. Operationally, time zero was set as the later of the following: the first documentation that the patient met the Sepsis-3 septic shock criteria: (i) lactate > 2.0 mmol/L, and (ii) initiation of vasopressor therapy, because both components are required to fulfill the definition. For each patient, Max NEE was identified as the highest NEE recorded during the observation period. The recorded time points included septic shock diagnosis (0 h), the time Max NEE was reached, the time Max NEE ended, and the times at which NEE decreased to 75%, 50%, 25%, and 0%. Based on these time points, Max NEE duration and the subsequent course of vasopressor dose reduction were calculated.

### 2.7. Norepinephrine Equivalent (NEE)

NEE was used to standardize the quantification of vasopressor dosing [9]. NEE was calculated using the following formula: norepinephrine (NE) dose (μg/kg/min) + epinephrine (Epi) dose (μg/kg/min) + 0.01 × dopamine (DoA) dose (μg/kg/min) + 0.06 × phenylephrine dose (μg/kg/min) + 2.5 × vasopressin (VA) dose (U/min) + 0.0025 × angiotensin II dose (ng/kg/min) + 10 × terlipressin dose (μg/kg/min) + 0.2 × methylene blue dose (mg/kg/h) + 1/8 × metaraminol dose (μg/kg/min) + 0.02 × hydroxocobalamin dose (g) + 0.4 × midodrine dose (μg/kg/min). Several NEE conversion formulas have been proposed in previous studies [15,16,17], and the 2023 version used in this study was developed based on these reports [9].

### 2.8. Statistical Analysis

All statistical analyses were conducted using JMP Student Edition version 18.2.1 (SAS Institute Inc., Cary, NC, USA). Normality of continuous variables was assessed, and most were non-normally distributed. Therefore, continuous variables were presented as medians with interquartile ranges (IQRs) and compared between the two groups, appropriate and inappropriate, using the Mann–Whitney U test. Categorical variables were summarized as counts and percentages and compared using Fisher’s exact test. A two-sided *p* value < 0.05 was considered statistically significant. Missing values were present in some laboratory variables not directly related to the primary outcomes. The number of missing observations was small, and missingness mainly reflected measurements that were not performed, consistent with the retrospective study design. Because these missing data were considered unlikely to be associated with the primary outcomes, a complete-case analysis was performed, and cases with missing values were excluded from the corresponding analyses. Given the exploratory nature of this retrospective study and the limited sample size, multivariable adjustment was not performed. Accordingly, the results were interpreted as unadjusted associations.

### 2.9. Ethics Approval and Consent to Participate

This single-center retrospective observational study used data obtained from an in-hospital database and patients’ medical records. The study protocol was approved by the Institutional Review Board of Nihon University Itabashi Hospital (approval number: RK-250114-1). Because of the retrospective design and exclusive use of existing data, the requirement for written informed consent was waived by the ethics committee. All data were anonymized before analysis. Study information was disclosed through an opt-out approach (e.g., institutional postings), and eligible patients were given the opportunity to decline participation.

## 3. Results

**Figure 1:**
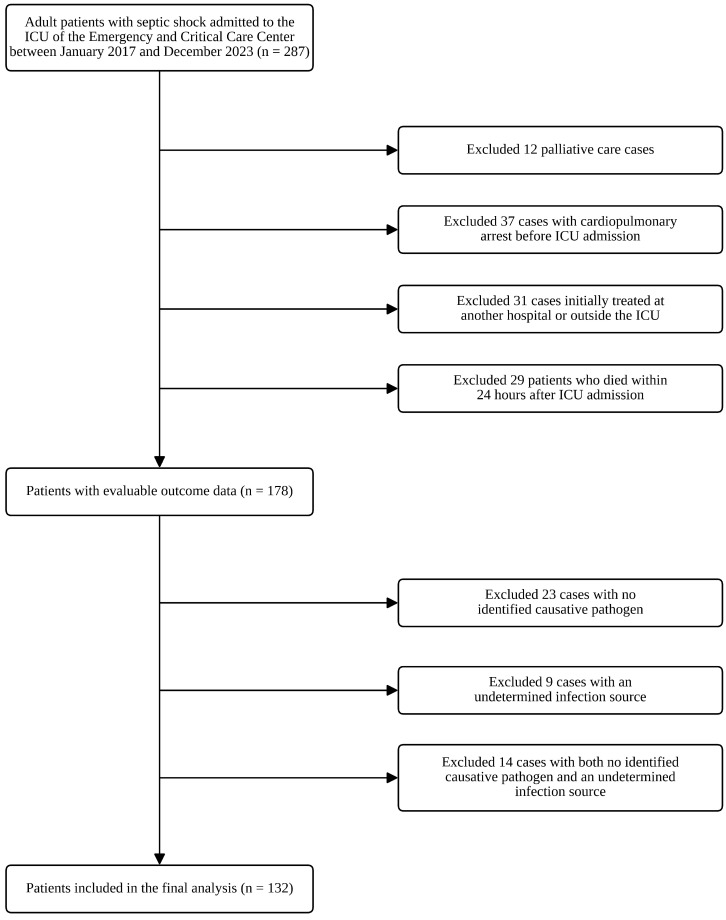
Patient selection flow diagram. Adult patients with septic shock admitted to the ICU of the Emergency and Critical Care Center between January 2017 and December 2023 were assessed for eligibility (*n* = 287). Of these, 12 patients receiving palliative care only, 37 with cardiopulmonary arrest before ICU admission, 31 in whom antibiotics and/or vasopressor therapy had already been initiated at another hospital or outside the ICU before ICU admission, and 29 who died within 24 h after ICU admission were excluded. Among the remaining 178 patients, 46 were excluded because the causative pathogen and/or infection source could not be identified: 23 with no identified causative pathogen, nine with an undetermined infection source, and 14 with both. Finally, 132 patients were included in the analysis. **Abbreviations:** ICU, intensive care unit.

The patient selection flow is shown in Figure 1. A total of 132 patients were included in the final analysis. Of these, 101 were classified into the appropriate group and 31 into the inappropriate group. Among the 31 patients in the inappropriate group, the causative pathogen was not covered by the initial antibiotic therapy in 11 patients, the presumed source of infection at the initial evaluation did not match the final infection source in 16 patients, and four patients met both criteria.

Baseline clinical characteristics are summarized in Table 1. ICU survival was observed in 61 patients (60%) in the appropriate group and 17 patients (55%) in the inappropriate group (*p* = 0.6771). Between-group differences were observed in the coagulation component of the SOFA score, 1 (0–2) vs. 0 (0–1) (*p* = 0.0134), the neutrophil-to-lymphocyte ratio, 19 (10–46) vs. 14 (7–24) (*p* = 0.0425), and chronic kidney disease, 8 (8%) vs. 7 (23%) (*p* = 0.0461). No other baseline characteristics differed significantly between the groups.

Infection-related parameters, including infection source, culture results, and antibiotic-related variables, are summarized in Table 2. Significant between-group differences were observed in skin and soft tissue infection as the infection source (9 [9%] vs. 9 [29%], *p* = 0.0131), positive blood cultures (68 [67%] vs. 29 [94%], *p* = 0.0044), positive bile cultures (14 [14%] vs. 0 [0%], *p* = 0.0396), GPC-positive blood cultures (20 [20%] vs. 17 [55%], *p* = 0.0004), negative blood cultures (33 [33%] vs. 2 [6%], *p* = 0.0044), and patients with resistant organisms (16 [16%] vs. 11 [36%], *p* = 0.0234). Among resistant organism profiles, methicillin-resistant Staphylococcus aureus (MRSA) was identified in 8 patients (8%) in the appropriate group and 7 patients (23%) in the inappropriate group, and methicillin-resistant coagulase-negative staphylococci (MRCNS) was identified in 0 patients (0%) and 2 patients (7%), respectively. Individual resistant organism types were presented descriptively.

Vasopressor dosing parameters expressed as NEE are summarized in Table 3. The vasopressor agents used were NE, VA, DoA, and Epi. At Max NEE, the dose of each agent was as follows (median [interquartile range]): NE, 0.31 (0.20–0.40) μg/kg/min; VA, 0 (0–0.052) U/min; DoA, 0 (0–0) μg/kg/min; and Epi, 0 (0–0) μg/kg/min. There were no significant differences between the appropriate and inappropriate groups in Max NEE or in the doses of the individual vasopressor agents at Max NEE. The time from septic shock diagnosis (time zero) to reaching Max NEE was significantly shorter in the appropriate group than in the inappropriate group (6 (2–12) h vs. 11 (5–17) h, *p* = 0.0177). Similarly, the time from septic shock diagnosis to the end of the Max NEE period was shorter in the appropriate group (13 (7–22) h vs. 21 (14–34) h, *p* = 0.0034).

### 3.1. Primary Outcome (1): Max NEE Duration

**Figure 2:**
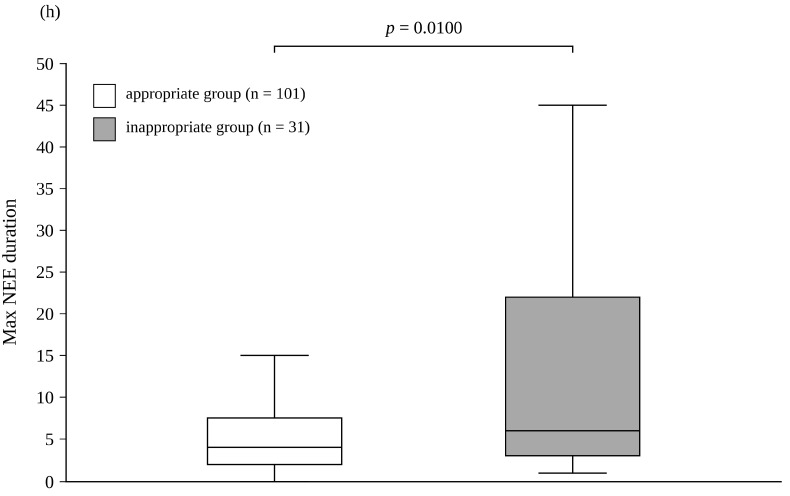
Max NEE duration in the appropriate and inappropriate groups. Max NEE duration was defined as the period during which the highest NEE was maintained. Data are presented as median (interquartile range). The Mann–Whitney U test was used for statistical comparisons. **Abbreviations:** DoA, dopamine; Epi, epinephrine; NE, norepinephrine; NEE, norepinephrine equivalent; VA, vasopressin.

Figure 2 shows Max NEE duration, defined as the period during which the highest NEE was maintained, in the appropriate and inappropriate groups. Max NEE duration was 4 (2–7) h in the appropriate group and 6 (3–20) h in the inappropriate group (*p* = 0.0100). During the Max NEE period, 13 patients died, 10 in the appropriate group and three in the inappropriate group. A sensitivity analysis restricted to patients who survived the Max NEE period was performed after excluding patients who died during the Max NEE period (*n* = 119). In this analysis, Max NEE duration was 6 (2–11) h in the appropriate group (*n* = 91) and 11 (5–14) h in the inappropriate group (*n* = 28) (*p* = 0.0314).

### 3.2. Primary Outcome (2): Time to NEE Reduction from Max NEE

**Figure 3:**
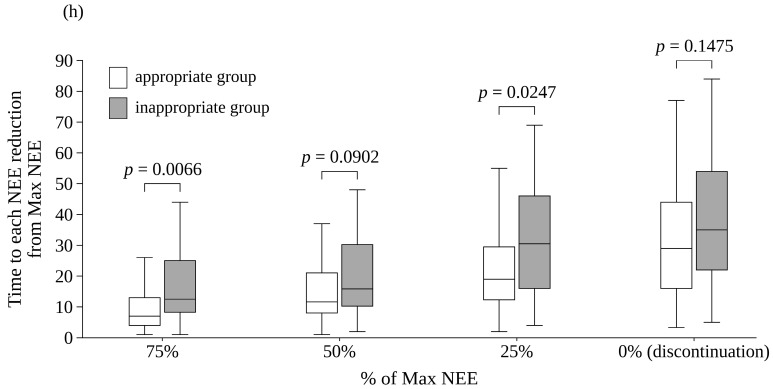
Time to each NEE reduction threshold from Max NEE in the appropriate and inappropriate groups. This figure shows the times required for NEE to decrease from the time Max NEE was reached to 75%, 50%, 25%, and 0% (discontinuation) in the appropriate and inappropriate groups. Each time (75%, 50%, 25%, and 0%) was calculated from the time at which NEE first reached Max NEE. Data are presented as median (interquartile range). The Mann–Whitney U test was used for statistical comparisons.

Figure 3 shows the time required for NEE to decrease from Max NEE to 75%, 50%, 25%, and 0% (discontinuation). Time to 75% was 7 (4–13) h in the appropriate group and 13 (9–23) h in the inappropriate group (*p* = 0.0066). Time to 50% was 12 (8–21) h vs. 16 (11–29) h (*p* = 0.0902). The time to 25% was 19 (13–29) h vs. 31 (16–46) h (*p* = 0.0247). Time to 0% (discontinuation) was 29 (16–44) h vs. 35 (23–53) h (*p* = 0.1475). The numbers of patients evaluated at each time point were 91 vs. 24 at 75%, 90 vs. 24 at 50%, 88 vs. 24 at 25%, and 87 vs. 23 at discontinuation. The decrease in the number of patients over time was attributable to deaths during vasopressor therapy.

## 4. Discussion

In patients with septic shock, the appropriate group, defined as patients in whom the causative pathogen was covered by initial antibiotic therapy and the presumed infection source was concordant with the final infection source, showed a shorter Max NEE duration and earlier vasopressor dose reduction. Prolonged Max NEE duration or delayed vasopressor dose reduction may reflect delayed clinical improvement and may help prompt bedside reassessment of initial antibiotic therapy and the presumed infection source. These findings should be interpreted as exploratory associations in a selected retrospective cohort, rather than evidence of independent predictive or causal utility.

Regarding the appropriateness of initial antibiotic therapy, Kumar et al. reported that mortality increases with each 1-h delay in administration of effective antibiotics [6]. Because no widely available test can reliably determine the adequacy of initial antibiotic coverage within the first several hours to 24 h, empiric antibiotics are administered in sepsis based on the pathogens most likely to be inferred from the clinical context [2]. As one potential approach to earlier assessment, rapid diagnostic methods such as multiplex PCR can enable early identification of causative pathogens and, in some assays, selected resistance determinants. However, the adequacy of antibiotic coverage cannot be fully established when PCR results are negative or when the causative organism is not included in the assay target panel. Van de Groep et al. reported that, among patients with sepsis requiring ICU admission, blood PCR results were negative in 48% of cases [18]. In addition, multiplex PCR is costly and has not been implemented in many institutions. Therefore, given its cost and limited coverage, multiplex PCR is not yet a universally applicable test across all healthcare settings.

Delays in source control are also linked to worse outcomes in sepsis. In septic shock caused by gastrointestinal perforation, the time from hospital admission to initiation of surgery for source control has been identified as a key determinant of survival [19]. In addition, inaccurate identification of the presumed infection source has been associated with worse ICU outcomes. In sepsis, patients in whom the presumed infection source at the initial evaluation did not match the final confirmed source had higher in-hospital mortality [7]. In patients with sepsis admitted to the ICU, an unknown infection source has been associated with a higher incidence of multiple organ failure [20]. In this study, invasive source control procedures tended to be performed less often in the inappropriate group than in the appropriate group, although the difference was not statistically significant. This finding may reflect difficulties in selecting or implementing invasive source control when the presumed infection source at the initial evaluation is discordant with the final infection source or remains uncertain. Taken together, prior literature and the findings of this study underscore the importance of identifying the infection source as early as possible, promptly re-evaluating the presumed infection source, and pursuing additional diagnostic work-up as needed during the clinical course of sepsis management. However, among patients with suspected sepsis, the infection source cannot be identified in up to 33% of cases [21]. Moreover, even among hospitalized patients diagnosed with sepsis according to the Sepsis-3 criteria, some ultimately have no confirmed infection, whereas in others the presumed infection source is revised during the clinical course [22]. Cases with an unknown infection source may include a proportion of non-infectious conditions that mimic sepsis (“sepsis mimics”); one report indicated that approximately 25% of patients initially presumed to have sepsis at ICU admission were ultimately diagnosed with sepsis mimics [23]. Therefore, in clinical practice, when the maximum vasopressor dose is prolonged, or vasopressor dose reduction is delayed, clinicians may need not only to reassess the adequacy of initial antibiotic coverage and the presumed infection source, but also to reconsider the differential diagnosis of non-infectious conditions, including sepsis mimics.

At present, readily available bedside information that can complement early reassessment of both initial antibiotic coverage and infection source evaluation remains limited. Kuwana et al. reported that, in patients with septic shock due to bacteremia, more rapid vasopressor dose reduction was associated with susceptibility to initial antibiotic therapy [10]. However, patients without bacteremia were excluded, and the adequacy of source control was not evaluated. In this study, Max NEE duration was shorter in the appropriate group than in the inappropriate group, despite a trend toward higher SOFA scores at ICU admission in the appropriate group, suggesting greater baseline organ dysfunction. Moreover, in a sensitivity analysis restricted to patients who survived the Max NEE period after exclusion of patients who died during the Max NEE period, Max NEE duration remained shorter in the appropriate group. Patients who died during the Max NEE period may have died primarily because of profound shock severity itself rather than because of problems related to initial antibiotic therapy or source control. Taken together, shorter maximum vasopressor duration and earlier vasopressor dose reduction were associated with adequate initial antibiotic coverage and infection source concordance in this selected cohort. These findings suggest that vasopressor dosing trajectories may reflect early clinical response and may help prompt bedside reassessment, but they should not be interpreted as validated indicators of treatment appropriateness or source control adequacy.

### Clinical Implications

In clinical practice, a prolonged need for high-dose vasopressor support after septic shock onset may provide a bedside cue to reassess both antibiotic coverage and the presumed infection source. For example, in this cohort, cases with a Max NEE duration exceeding 12 h and/or requiring more than 24 h for vasopressor dose reduction to 25% of the maximum dose were more common in the inappropriate group. These examples are intended to reflect the observed between-group separation in Max NEE duration and vasopressor tapering within this cohort. These timeframes should be interpreted as exploratory reference points for bedside reassessment rather than universal cutoffs, because vasopressor trajectories may be influenced by institution-specific resuscitation practices and patient severity. Under such circumstances, reassessment of antibiotic therapy, renewed evaluation of the infection source, and timely consideration of source control may be appropriate. This approach is intended to support timely clinical vigilance rather than mandate a fixed time-based decision threshold.

In particular, the microbiological profile observed in the inappropriate group provides a clinically relevant context for early reconsideration of empiric antibiotic coverage. In the inappropriate group, the proportion of patients with GPC-positive blood cultures was higher, and MRSA and MRCNS, pathogens that typically require anti-MRSA agents, were identified more frequently. In a large-scale study of bloodstream infections, MRSA as the causative pathogen was associated with an approximately nine-fold higher risk of inappropriate initial antibiotic therapy than non-MRSA pathogens [5]. However, because anti-MRSA agents increase the risk of nephrotoxicity, routine empiric anti-MRSA coverage for all patients with sepsis is not recommended in sepsis guidelines. Instead, the SSC guidelines recommend considering empiric anti-MRSA therapy based on patient-specific MRSA risk factors, such as prior history, healthcare-associated exposures, and local MRSA prevalence [2]. Accordingly, in patients with septic shock who are not initially treated with anti-MRSA agents, prolonged maximum vasopressor duration and delayed vasopressor dose reduction may prompt reassessment of MRSA/MRCNS risk and the adequacy of empiric antibiotic coverage, while taking the patient’s MRSA risk profile into account. However, these microbiological differences also represent an important baseline imbalance and potential confounder when interpreting the association between vasopressor trajectories and the composite classification of initial antibiotic coverage and infection source concordance.

This study has some limitations. First, this was a single-center retrospective observational study in which initial antibiotic selection and vasopressor dosing were determined by clinical judgment, and patients were subsequently classified retrospectively on that basis. Therefore, selection bias related to differences in patient characteristics and treatment strategies, as well as the influence of unmeasured confounders, cannot be excluded. Because multivariable adjustment was not performed, the observed differences should be interpreted as unadjusted associations rather than causal effects. Although baseline imbalances were observed in chronic kidney disease, skin and soft tissue infection, and microbiological profiles such as GPC-positive blood cultures and MRSA/MRCNS, multivariable adjustment or propensity score matching was not performed because of the limited sample size and the small number of patients in the inappropriate group. Therefore, these clinical and microbiological imbalances may have confounded the observed associations between the composite classification and vasopressor dosing trajectories. Patients who died within 24 h were excluded because vasopressor dosing trajectories could not be meaningfully evaluated; however, this may limit applicability to fulminant septic shock and may have introduced survivorship bias by excluding the most severely ill patients. Prospective studies are needed to validate these associations and clarify their clinical utility. Second, 46 patients (25.8%) in whom either the causative pathogen or the infection source remained unidentified were excluded. Accordingly, the generalizability of these findings is limited to patients in whom both the causative pathogen and infection source were identified. Therefore, it remains uncertain whether the associations between vasopressor dosing duration and initial antibiotic coverage or infection source concordance can be extrapolated to populations that include patients with an unknown causative pathogen or infection source. In addition, the appropriate/inappropriate classification was based on a composite measure, pathogen susceptibility to initial antibiotic therapy and accuracy of presumed source identification, so the relative contribution of each component to vasopressor trajectories could not be determined in this study. Because the final infection source was determined using information available up to ICU discharge, prolonged vasopressor requirement may have led to additional diagnostic evaluation and subsequent reclassification of the initially presumed infection source; therefore, the temporal direction of this association could not be fully established. Future studies with larger sample sizes are warranted to evaluate these components separately. Third, baseline microbiological imbalance may have influenced the observed vasopressor trajectories. The inappropriate group had different infection and microbiological profiles, including higher frequencies of GPC-positive blood cultures and resistant organisms such as MRSA/MRCNS. These factors may affect the timing of effective antibiotic coverage, diagnostic confirmation, source control requirements, and hemodynamic response. Therefore, the observed associations cannot be attributed solely to the composite classification of initial antibiotic coverage and infection source concordance. Fourth, although hemodynamic management and antibiotic therapy were generally guided by standard practice and clinical guidelines, variability in therapeutic interventions cannot be excluded, including differences in vasopressor selection, administered fluid volume, corticosteroid use and timing, timing of antibiotic escalation or modification, and adequacy of source control. In addition, patient-level pharmacokinetic/pharmacodynamic target attainment could not be evaluated because measured antibiotic concentrations were not available in this retrospective study. These factors may have influenced vasopressor dosing trajectories. In addition, death during vasopressor therapy precluded observation of subsequent NEE reduction milestones, introducing informative censoring into the time-course outcomes. Although a sensitivity analysis restricted to survivors was performed, this approach may introduce survivor bias; therefore, the findings regarding time to NEE reduction thresholds should be interpreted cautiously. In addition, no significant difference in ICU survival was observed between the appropriate and inappropriate groups, and, because of the retrospective design, 28-day survival could not be adequately ascertained in many patients. Missing values in some laboratory variables were handled using complete-case analysis, which may also have introduced bias, although these variables were not directly related to the primary outcomes. Therefore, this study alone cannot determine whether interventions guided by vasopressor dosing duration would improve survival outcomes. Future prospective studies with predefined treatment protocols and systematic follow-up are needed to evaluate the impact of such interventions on clinical outcomes. In addition, future studies should evaluate whether vasopressor dosing trajectories can be integrated with emerging molecular biomarkers of sepsis to support early bedside risk stratification and precision management in septic shock [24].

## 5. Conclusions

In patients with septic shock, the appropriate group, defined as patients in whom the causative pathogen was covered by initial antibiotic therapy and the presumed infection source was concordant with the final infection source, showed a shorter Max NEE duration and earlier vasopressor dose reduction. Prolonged Max NEE duration and delayed NEE reduction were associated with inadequate initial antibiotic coverage or discordant infection source identification. Vasopressor dosing trajectories may help prompt bedside reassessment of antibiotic therapy and infection source evaluation.

## Figures and Tables

**Table 1:** Clinical characteristics of patients in the appropriate and inappropriate groups.

		All	Appropriate Group	Inappropriate Group	*p* Value
	Number	132	101	31	
	Age	79 (71–85)	79 (71–85)	85 (73–90)	0.9422
	Male	77 (58%)	56 (55%)	21 (67%)	0.3142
	BMI	21 (18–24)	21 (18–24)	21 (17–24)	0.8091
	Weight (kg)	52 (44–63)	52 (44–63)	52 (43–63)	0.9316
Outcome	ICU survival	78 (59%)	61 (60%)	17 (55%)	0.6771
	ICU length of stay (days)	6 (3–13)	7 (4–13)	4 (3–10)	0.2115
	28-day survival	64 (60%) (missing; 26)	51 (64%) (missing; 21)	13 (50%) (missing; 5)	0.3103
Patient profile	SOFA score	12 (9–15)	13 (9–15)	11 (10–13)	0.0870
	Respiration	2 (2–3)	2 (1–3)	2 (2–3)	0.9237
	Coagulation	1 (0–2)	1 (0–2)	0 (0–1)	0.0134
	Liver	0 (0–1)	0 (0–1)	0 (0–1)	0.0941
	Cardiovascular	4 (4–4)	4 (4–4)	4 (4–4)	0.7410
	Central nervous system	2 (1–3)	2 (1–3)	2 (1–2)	0.8095
	Renal	2 (1–3)	2 (1–3)	2 (1–4)	0.5959
	Initial lactate (mmol/L)	5.0 (3.3–7.2)	5.1 (3.4–7.2)	4.4 (2.7–6.5)	0.2169
	Initial pH	7.40 (7.28–7.44)	7.35 (7.27–7.44)	7.35 (7.30–7.42)	0.9914
	WBC (10^4^/µL)	1.1 (0.6–1.8)	1.2 (0.6–2.0)	1.1 (0.6–1.3)	0.3919
	Neutrophil-to-lymphocyte ratio	18 (9–37) (missing; 5)	19 (10–46) (missing; 5)	14 (7–24) (missing; 0)	0.0425
	CRP (mg/dL)	13 (4–25)	14 (4–26)	9 (4–19)	0.2977
Healthcare exposure and risk factors for MDR organisms	Acquisition setting				
	- Community-acquired	97 (73%)	75 (74%)	22 (71%)	0.8165
	- Healthcare-associated	22 (17%)	16 (16%)	6 (19%)	0.7831
	- Hospital-acquired	13 (10%)	10 (10%)	3 (10%)	1.0000
	Hospitalization for ≥2 days within the previous 90 days	18 (14%)	15 (15%)	3 (10%)	0.5630
	Prior antibiotic exposure within the previous 90 days	8 (6%)	7 (7%)	1 (3%)	0.6800
Comorbidities	Diabetes mellitus	52 (39%)	42 (42%)	10 (32%)	0.4054
	Chronic pulmonary disease	10 (8%)	8 (8%)	2 (6%)	1.0000
	Chronic liver disease	20 (15%)	16 (16%)	4 (13%)	0.7828
	Chronic kidney disease	15 (11%)	8 (8%)	7 (23%)	0.0461
	Chronic heart failure	42 (32%)	31 (31%)	11 (35%)	0.6620
	Immunosuppression ^a^	12 (9%)	7 (7%)	5 (16%)	0.1516
	Malignancy	30 (23%)	23 (23%)	7 (23%)	1.0000
Treatment	Mechanical ventilation	85 (64%)	66 (65%)	19 (61%)	0.8429
	Renal replacement therapy	45 (34%)	31 (31%)	14 (45%)	0.2041
	Low-dose corticosteroids	106 (80%)	81 (80%)	25 (81%)	1.0000
	Total fluid volume administered during the first 24 h (mL)	6687 (5358–8967)	6973 (5300–10,255)	6423 (5451–7823)	0.2110
	ECMO	0	0	0	
	Invasive source control procedures ^b^	42 (32%)	37 (37%)	5 (16%)	0.0544
	- Drainage/decompression	31 (24%)	28 (28%)	3 (10%)	
	- Surgery/resection/repair	10 (8%)	8 (8%)	2 (7%)	
	- Debridement/amputation	3 (2%)	3 (3%)	0 (0%)	
	- Device removal/exchange	0	0	0	

**Notes:** ^a^ Immunosuppression comprised systemic corticosteroid use (appropriate group, *n* = 7; inappropriate group, *n* = 5), immunosuppressive therapy (appropriate group, *n* = 2; inappropriate group, *n* = 1), or antineoplastic therapy (appropriate group, *n* = 1; inappropriate group, *n* = 0). Some patients met more than one criterion: overlapping cases were present in 2 patients in the appropriate group and 1 patient in the inappropriate group. ^b^ Two patients underwent multiple source control procedures (appropriate group, *n* = 2; inappropriate group, *n* = 0). Individual source control measures are presented descriptively. *p* value, appropriate vs. inappropriate. Data are presented as median (interquartile range) or *n* (%). **Abbreviations:** BMI, body mass index; CRP, C-reactive protein; ECMO, extracorporeal membrane oxygenation; ICU, intensive care unit; MDR, multidrug-resistant; SOFA, Sequential Organ Failure Assessment; WBC, white blood cell.

**Table 2:** Infection-related parameters in the appropriate and inappropriate groups.

		All	Appropriate Group	Inappropriate Group	*p* Value
	Number	132	101	31	
Infection source ^a^	UTI	43 (33%)	34 (34%)	9 (29%)	0.7932
	Pneumonia	34 (26%)	28 (28%)	6 (19%)	0.4857
	Intra-abdominal infection	21 (16%)	18 (18%)	3 (10%)	0.4215
	Biliary tract infection	19 (14%)	17 (17%)	2 (6%)	0.2511
	Skin and soft tissue infection	18 (14%)	9 (9%)	9 (29%)	0.0131
	Others ^b^	5 (4%)	2 (2%)	3 (10%)	0.1539
Positive culture specimens ^c^	Blood	97 (73%)	68 (67%)	29 (94%)	0.0044
	Sputum	42 (32%)	33 (33%)	9 (29%)	0.8267
	Urine	69 (52%)	53 (52%)	16 (52%)	1.0000
	Bile	14 (11%)	14 (14%)	0 (0%)	0.0396
	Abscess/pus	12 (9%)	9 (9%)	3 (10%)	1.0000
	Ascites	11 (8%)	10 (10%)	1 (3%)	0.4570
	Soft tissue	1 (1%)	1 (1%)	0 (0%)	1.0000
	Joint fluid	1 (1%)	1 (1%)	0 (0%)	1.0000
Gram stain classification of blood culture ^d^	GPC	37 (28%)	20 (20%)	17 (55%)	0.0004
	GPR	15 (11%)	13 (13%)	2 (6%)	0.5191
	GNC	0 (0%)	0 (0%)	0 (0%)	
	GNR	61 (46%)	47 (47%)	14 (45%)	1.0000
	Negative	35 (27%)	33 (33%)	2 (6%)	0.0044
Initial antibiotics ^e^	ABPC/SBT	9	6	3	
	PIPC/TAZ	51	42	9	
	CMZ	4	4	0	
	CTRX	19	12	7	
	CFPM	19	14	5	
	MEPM	15	12	3	
	DRPM	9	8	1	
	VCM	2	1	1	
	TEIC	26	20	6	
	Others ^f^	7	5	3	
Time from diagnosis to initial antibiotic administration (min)		68.0 (43–122)	64.0 (41–111)	84.0 (58.5–143)	0.2009
De-escalation		61 (46%)	45 (45%)	16 (52%)	0.5404
Duration of antibiotic therapy (days)		11 (4–15)	11 (4–15)	9 (3.5–15.5)	0.8782
Patients with resistant organisms		27 (21%)	16 (16%)	11 (36%)	0.0234
Resistant organism type ^g^	ESBL-producing Enterobacterales	11 (8%)	8 (8%)	3 (10%)	-
	AmpC-producing Enterobacterales	1 (1%)	1 (1%)	0 (0%)	-
	Carbapenem- resistant Enterobacterales	0 (0%)	0 (0%)	0 (0%)	-
	MDR-PA	0 (0%)	0 (0%)	0 (0%)	-
	MRSA	15 (11%)	8 (8%)	7 (23%)	-
	MRCNS	2 (2%)	0 (0%)	2 (7%)	-
	VRE	0 (0%)	0 (0%)	0 (0%)	-

**Notes:** ^a^ Includes 8 cases with multiple infection sources (appropriate group, *n* = 7; inappropriate group, *n* = 1). ^b^ Other sources included septic arthritis (*n* = 2), infective endocarditis (*n* = 2) and pyometra (*n* = 1). ^c^ Includes patients with one or more positive culture specimens; categories were not mutually exclusive. ^d^ Includes 13 polymicrobial cases (appropriate group, *n* = 10; inappropriate group, *n* = 3). ^e^ Initial antibiotic therapy: single-agent therapy in 102 cases and combination therapy (≥2 antibiotics) in 30 cases. ^f^ Other antibiotics: CLDM (*n* = 3), CAZ (*n* = 2), CTX (*n* = 1), LVFX (*n* = 1), and MNZ (*n* = 1), including one overlapping case. ^g^ Includes 2 cases with more than one resistant organism type (appropriate group, *n* = 1; inappropriate group, *n* = 1). Individual resistant organism types are presented descriptively. *p* value, appropriate vs. inappropriate. Data are presented as median (interquartile range) or *n* (%). **Abbreviations:** AmpC, AmpC β-lactamase; ESBL, extended-spectrum β-lactamase; GNC, Gram-negative cocci; GNR, Gram-negative rods; GPC, Gram-positive cocci; GPR, Gram-positive rods; MDR-PA, multidrug-resistant Pseudomonas aeruginosa; MRCNS, methicillin-resistant coagulase-negative staphylococci; MRSA, methicillin-resistant Staphylococcus aureus; UTI, urinary tract infection; VRE, vancomycin-resistant enterococci. **Antibiotic abbreviations:** ABPC/SBT, ampicillin/sulbactam; CAZ, ceftazidime; CFPM, cefepime; CLDM, clindamycin; CMZ, cefmetazole; CTRX, ceftriaxone; CTX, cefotaxime; DRPM, doripenem; LVFX, levofloxacin; MEPM, meropenem; MNZ, metronidazole; PIPC/TAZ, piperacillin/tazobactam; TEIC, teicoplanin; VCM, vancomycin.

**Table 3:** NEE parameters in the appropriate and inappropriate groups.

		All	Appropriate Group	Inappropriate Group	*p* Value
	Number	132	101	31	
NEE	Max NEE	0.37 (0.21–0.45)	0.37 (0.22–0.45)	0.38 (0.21–0.45)	0.6271
	Max NE ^1^	0.31 (0.20–0.40)	0.31 (0.19–0.39)	0.34 (0.21–0.40)	0.5585
	Max VA ^2^	0 (0–0.052)	0 (0–0.042)	0 (0–0.083)	0.7258
	Max DoA ^3^	0 (0–0)	0 (0–0)	0 (0–0)	0.1214
	Max Epi ^4^	0 (0–0)	0 (0–0)	0 (0–0)	0.2114
NEE time (h)	Time from septic shock diagnosis to reaching Max NEE	7 (3–14)	6 (2–12)	11 (5–17)	0.0177
	Time from septic shock diagnosis to the end of the Max NEE period	14 (7–26)	13 (7–22)	21 (14–34)	0.0034

**Notes:** *p* value, appropriate vs. inappropriate. Data are presented as median (interquartile range). ^1^ NE = 1 × NE (μg/kg/min), ^2^ VA = 2.5 × VA (U/min), ^3^ DoA = 0.01 × DoA (μg/kg/min), ^4^ Epi = 1 × Epi (μg/kg/min). **Abbreviations:** DoA, dopamine; Epi, epinephrine; NE, norepinephrine; NEE, norepinephrine equivalent; VA, vasopressin.

## Data Availability

The data presented in this study were fully anonymized to protect patient privacy. Due to institutional and ethical restrictions, the data are not publicly available. The anonymized dataset (including the Excel file used for the analyses) is available from the corresponding author upon request and with approval from the Institutional Review Board.

## Abbreviations

The following abbreviations are used in this manuscript:

BMI	Body mass index
DoA	Dopamine
Epi	Epinephrine
GNC	Gram-negative cocci
GNR	Gram-negative rods
GPC	Gram-positive cocci
GPR	Gram-positive rods
ICU	Intensive care unit
IQR	Interquartile range
MALDI-TOF MS	Matrix-assisted laser desorption/ionization time-of-flight mass spectrometry
MAP	Mean arterial pressure
MRCNS	Methicillin-resistant coagulase-negative staphylococci
MRSA	Methicillin-resistant *Staphylococcus aureus*
NE	Norepinephrine
NEE	Norepinephrine equivalent
PCR	Polymerase chain reaction
SOFA	Sequential Organ Failure Assessment
SSC	Surviving Sepsis Campaign
VA	Vasopressin
